# The new Flemings now sing: a methodological evaluation of gamification and citizen science strategies to raise awareness on antimicrobial resistance

**DOI:** 10.1111/imcb.70094

**Published:** 2026-02-13

**Authors:** Antonio Tarín‐Pelló, Sara Fernández‐Álvarez, Beatriz Suay‐García, Elisa Marco‐Crespo, José Ignacio Bueso‐Bordils, Carolina Galiana‐Roselló, María‐Teresa Pérez‐Gracia

**Affiliations:** ^1^ Área de Microbiología, Departamento de Farmacia, Instituto de Ciencias Biomédicas, Facultad de Ciencias de la Salud Universidad Cardenal Herrera‐CEU, CEU Universities Alfara del Patriarca Spain; ^2^ Departamento de Matemáticas, ESI International Chair@CEU‐UCH, Física y Ciencias Tecnológicas Universidad Cardenal Herrera‐CEU, CEU Universities Alfara del Patriarca Spain; ^3^ Unidad de Cultura Científica, Departamento de Comunicación e Información Periodística Universidad Cardenal Herrera‐CEU Alfara del Patriarca Spain

**Keywords:** Antimicrobial resistance, awareness, citizen science, health education, public engagement, science communication

## Abstract

Antimicrobial resistance (AMR) represents one of the greatest global health threats of the 21st century, yet public understanding of this silent pandemic remains limited. This study presents the design, implementation and evaluation of the SWICEU project, developed at CEU Cardenal Herrera University (Spain), which integrates citizen science, gamification and creative communication strategies to raise awareness of AMR and promote scientific vocations and the responsible use of antibiotics among young people and the general population. Over eight editions (2017–2025), 287 university students and 853 pre‐university students aged between 14 and 16 participated in activities combining experimental microbiology with creative formats such as storytelling, music, board games and digital campaigns to promote becoming the Flemings of the future and raise awareness of AMR. The project's impact was assessed through pre‐ and post‐surveys, satisfaction measures and analytics from media and social platforms including YouTube, X and Instagram. Results showed significant knowledge gains among participants (from 68.8% to 80.9% correct responses, *P* < 0.05), high satisfaction ratings (4.86–5/5) and wide public engagement, reaching more than 3.5 million people through media coverage and over 819 000 impressions on social media. These findings demonstrate that combining citizen participation, gamified learning and creative media strategies can make complex microbiological concepts both accessible and engaging. The SWICEU model provides a transferable framework for enhancing science communication and health literacy, not only in AMR education but also across other public health areas requiring societal awareness and behavior change.

## Graphical abstract

The SWICEU project's awareness strategy against the rise of AMR combines direct participation in laboratory experiments and awareness campaigns as well as the creation of gamification materials to engage the public. All of this is supported by dissemination through the media, social networks and participation in online and live events.

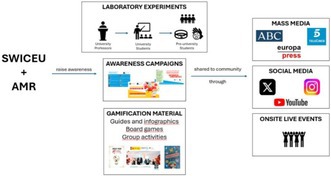

## INTRODUCTION

Antimicrobial resistance (AMR) is one of the most urgent global public health threats of the 21st century.^1^ Defined by the World Health Organization (WHO) as a silent pandemic, AMR was directly responsible for 1.27 million deaths in 2019 and contributed to nearly five million more worldwide.^2^ In Spain, the Spanish Society of Infectious Diseases and Clinical Microbiology (SEIMC) estimates that infections caused by resistant microorganisms lead to more than 23 000 deaths annually, placing Spain among the most affected countries in Europe.^3^ This global trend threatens to reverse decades of progress in medicine, jeopardizing routine surgery, cancer therapy and other treatments that depend on effective antimicrobials.^4^

The WHO recognizes AMR among the 10 leading global health threats, and the European Commission warns that antibiotic resistance could cause 10 million deaths per year by 2050 if no effective countermeasures are implemented.^4^ Despite its magnitude, public awareness remains limited.^5^ Studies indicate that many citizens confuse bacterial resistance with the human body's immunity or believe antibiotics are effective against viral infections.^6^ These misunderstanding fuels behaviors such as self‐medication, incomplete treatments and pressure on clinicians to prescribe antibiotics unnecessarily, all of which accelerate the emergence of resistant strains.

In parallel, public interest in science and health topics is increasing. The 2024 Survey on Social Perception of Science and Technology in Spain, conducted by the Spanish Foundation for Science and Technology (FECYT), reports that “Medicine and Health” is the area of greatest citizen interest (mean score 3.68/5), surpassing topics such as Food and Environment.^7^ However, only 4.5% of respondents feel “very well informed,” while two‐thirds acknowledge limited knowledge of these issues, revealing a significant gap between interest (3.68/5) and perceived information (3.05/5). This disconnect highlights the need for innovative science communication strategies that convert curiosity into understanding.

Mistrust toward scientific information remains another critical barrier. According to the same FECYT report, 50% of Spanish citizens believe that pharmaceutical companies hide vaccine risks and 42% suspect viruses are artificially created in laboratories. Similar findings appear across Europe: the 2025 Eurobarometer on Science and Technology shows that 36% of respondents cite lack of scientific knowledge as a barrier to engagement and 58% wish to be better informed.^8^ Nevertheless, most Europeans (77%) still believe biomedical research and infection control will positively impact life quality in the next two decades.

This paradox, high interest but low understanding and trust, underscores the urgent need for more engaging, participatory and emotionally resonant approaches to science education and health communication.^9^ Traditional, top‐down dissemination has proven insufficient to promote behavioral change. Conversely, participatory and experiential approaches, particularly those grounded in gamification and citizen science, enhance comprehension and foster sustainable behavioral shifts.^10^ Gamification increases motivation and conceptual retention through game dynamics, while citizen science initiatives build trust by involving non‐experts in real research, enhancing both scientific literacy and social responsibility.^11^

In this context, the SWICEU project, promoted by CEU Cardenal Herrera University (Valencia, Spain), was created in 2017 as an interdisciplinary initiative combining citizen science, educational innovation and creative communication to raise awareness about AMR^12^ and promote scientific vocations. Inspired by international programs such as Tiny Earth and integrated into the MicroMundo network, SWICEU engages pre‐university and university students in the search for new antibiotic‐producing microorganisms from soil samples.^13^, ^14^ Through this authentic research experience, participants become “the new Flemings,” symbolically recreating the scientific discovery that revolutionized modern medicine.

However, SWICEU extends well beyond the laboratory. It constitutes a multidisciplinary ecosystem that merges citizen science, gamification and media outreach to translate microbiological complexity into relatable and memorable learning experiences. Activities range from board games and escape rooms to microstory writing, digital challenges and musical performances, each designed to foster motivation and comprehension while countering misinformation.^15^, ^16^ These playful strategies transform abstract content (e.g. mutation, resistance mechanisms, natural selection) into concrete, emotionally engaging experiences that facilitate learning and retention.

However, SWICEU extends well beyond the laboratory. It constitutes a multidisciplinary ecosystem that merges citizen science, gamification and media outreach to translate microbiological complexity into relatable and memorable learning experiences. Activities range from board games and escape rooms to microstory writing, digital challenges and musical performances, each designed to foster motivation and comprehension while countering misinformation.^15^, ^16^ These playful strategies transform abstract content (e.g. mutation, resistance mechanisms, natural selection) into concrete, emotionally engaging experiences that facilitate learning and retention.

SWICEU's gamified model serves a dual purpose: first, to attract younger audiences accustomed to interactive digital environments, and second, to counter misinformation through creative, evidence‐based narratives. The combination of experimentation, creativity and art not only enhances cognitive understanding but also fosters emotional connection, a key determinant of long‐term attitude change toward antibiotic use.

This article presents a methodological and evaluative analysis of the SWICEU project. We describe the design, implementation and assessment of its educational and outreach activities, combining quantitative (knowledge tests, satisfaction surveys, social media analytics) and qualitative (participant reflections, feedback) approaches. By integrating empirical evaluation with creative communication, SWICEU demonstrates how participatory, gamified learning can contribute to public engagement with AMR and serve as a scalable model for other global health challenges where comprehension and behavioral change are essential.

## RESULTS

### Participation and scope

Across its eight editions (2017–2025), the SWICEU project engaged a total of 1140 participants, including 853 pre‐university students aged between 14 and 16 from seven secondary and high schools in Valencia and 287 university students from CEU Cardenal Herrera University.

University participants represented 11 degrees across three knowledge areas: Health Sciences: Pharmacy, Veterinary Medicine, Medicine, Nursing, Nutrition, Dentistry and Optometry; Communication Sciences: Journalism, Advertising and Public Relations and Audiovisual Communication; and Engineering and Design: Industrial Design Engineering and Product Development.

This multidisciplinary involvement enabled effective integration between scientific and communication perspectives, fostering a model of *science with and for society*.

### Scientific outcomes from citizen science

Pre‐university students contributed a total of 853 soil samples for analysis, from which 8530 microbial colonies were isolated under the supervision of university students. Of these isolates, 388 exhibited antibiosis activity, confirming potential antibiotic production.

These microbial strains have been preserved within the Microbiology Collection of the Pharmacy Department at CEU UCH, where they are available for further research and characterization by R&D teams. This tangible output exemplifies how citizen science can generate valuable scientific resources while simultaneously enhancing participants' engagement and understanding of microbiological processes.

### Educational impact: Knowledge and awareness

To assess the educational effect of the citizen science project, pre‐ and post‐intervention surveys were administered to all pre‐university participants.

Statistical analysis revealed a significant improvement in AMR‐related knowledge following participation. The overall percentage of correct answers increased from 68.8% (8.25/12) before the intervention to 80.9% (9.7/12) afterward, equivalent to an average improvement of 1.45 correct answers per student.

Differences were statistically significant (*P* < 0.05) in seven of the 12 questions, particularly those related to the need for prescriptions, proper antibiotic use, resistance mechanisms and the role of the WHO in global health (Table 1). These findings confirm that participation in the SWICEU laboratory component and gamification activities included in the project effectively strengthened students' conceptual understanding of AMR and the responsible use of antibiotics.

**Table 1:** Comparison of pre‐ and post‐test results among pre‐university students (*n* = 853 students).

Question	Start (%)	End (%)	*P* value*
Q1. What causes the common cold?	57.3	65.7	0.268
Q2. Is a prescription necessary to buy antibiotics?	67.1	87.3	**0.000** *
Q3. Which of the following medicines is an antibiotic?	76.4	89.3	**0.008** *
Q4. Are antibiotics useful for any type of infection?	76.9	84.0	**0.003** *
Q5. Can a bacterial infection be treated with any antibiotic?	60.1	75.9	**0.029** *
Q6. Which of these indications should be kept in mind when taking antibiotics?	66.1	78.8	0.072
Q7. If we do not take medications such as antibiotics well	92.1	95.7	0.076
Q8. What do you mean by antibiotic resistance?	67.3	79.7	**0.000** *
Q9. Antibiotic‐resistant bacteria can be transmitted to humans	74.8	76.6	0.564
Q10. Essential to combating antibiotic resistance is	49.9	64.9	**0.008** *
Q11. Indicate the erroneous statement	74.4	79.9	0.190
Q12. By 2050 and according to the World Health Organization (WHO), it is estimated that the first cause of death will be	80.2	92.8	**0.003** *
Overall	68.8	80.9	**0.000** *

Bold indicates statiscally significant (*P* < 0.05).

* Statistically significant at 95% confidence interval.

### Overall satisfaction survey

Participant satisfaction was consistently high across all eight editions. On a 1–5 scale, pre‐university students, university students and teachers from participating schools reported an average satisfaction score of 4.86, 4.94 and 5.00, respectively. These results indicate an overall positive perception of the program's educational value, organization and relevance to real‐world scientific issues.

### Gamification and creative outreach materials

Over the years, SWICEU has developed an extensive catalog of educational, recreational and gamified materials; all designed to make AMR concepts engaging and accessible to audiences of different ages and backgrounds (Table 2):

1. Informative, educational, and creative materials: the illustrated guide “Create Your Microstory”^17^ (3rd edition, Spanish/English) encourages students to craft short narratives, comics or videos inspired by microbes. It provides factual sheets on bacterial species and antibiotics, transforming scientific content into storytelling resources. The guide is complemented by multilingual infographics, translated into 20 languages, that summarize WHO‐based recommendations for responsible antibiotic use.
2. Board games: card games such as “2050: ¡Infección!” and Superbugs use microbiological characters and scenarios to introduce scientific terminology and processes through play. A more complex board game, “Hipótesis,” set in a university microbiology lab, uses a mystery format to teach key microbiological and epidemiological concepts.
3. Live events and immersive experiences: SWICEU's interactive activities include the campus‐based “Enigma on Campus” live Cluedo performance, the *portable Scape box* (team puzzle on AMR topics) and the “Become a Bact‐Hunter” QR‐based scavenger hunt. These activities combine entertainment with inquiry‐based learning.

**Table 2:** Educational and gamification materials and events (2017–2025).

Type of material	Description	Link
Illustrated guide Create Your Microstory^17^	Comprehensive creative guide integrating microbiological facts and narrative prompts to foster scientific creativity	https://medios.uchceu.es/actualidad‐ceu/microhistorias‐que‐conciencian‐ante‐un‐reto‐de‐salud‐global/
Multilingual infographics	10 key tips for appropriate antibiotic use (20 languages, WHO‐based)	https://medios.uchceu.es/actualidad‐ceu/diez‐consejos‐para‐hacer‐un‐buen‐uso‐de‐los‐antibioticos‐y‐frenar‐las‐resistencias‐bacterianas‐del‐equipo‐swiceu/
Card games “2050: ¡Infección!” and Superbugs	Educational decks linking roles and functions in microbiology and AMR	https://medios.uchceu.es/actualidad‐ceu/swiceu‐presenta‐sus‐ultimos‐formatos‐innovadores‐para‐la‐divulgacion‐de‐las‐resistencias‐bacterianas‐en‐la‐sociedad‐espanola‐de‐microbiologia/
Board game “Hipótesis”	Clue‐style board game about AMR set in a university environment	https://medios.uchceu.es/actualidad‐ceu/mas‐de‐cien‐escolares‐en‐el‐proyecto‐cientifico‐de‐busqueda‐de‐nuevos‐antibioticos‐con‐la‐ceu‐uch/
Scape box	Portable puzzle‐based learning game on AMR	https://medios.uchceu.es/actualidad‐ceu/swiceu‐presenta‐sus‐ultimos‐formatos‐innovadores‐para‐la‐divulgacion‐de‐las‐resistencias‐bacterianas‐en‐la‐sociedad‐espanola‐de‐microbiologia/
Live game Enigma on Campus	Street‐theater Cluedo experience on AMR	https://medios.uchceu.es/actualidad‐ceu/swiceu‐organiza‐un‐street‐scape‐divulgativo‐sobre‐el‐uso‐correcto‐de‐los‐antibioticos‐y‐el‐aumento‐de‐las‐infecciones‐bacterianas‐resistentes/
Become a Bact‐Hunter challenge	QR scavenger hunt for WHO priority bacteria	https://medios.uchceu.es/actualidad‐ceu/nuevo‐reto‐swiceu‐hazte‐bact‐hunter/
Karaoke “Resistencia en armonía”	Awareness activity through adapted and original music performances. Included original song “Resistencia en armonía”	https://medios.uchceu.es/actualidad‐ceu/un‐karaoke‐para‐concienciar‐sobre‐el‐aumento‐de‐las‐infecciones‐bacterianas‐resistentes‐a‐los‐antibioticos/

Music‐based outreach also played a key role: the “Resistencia en armonía” karaoke initiative involved adapting popular songs to promote awareness of AMR. Supported by CEU Música, this activity culminated in the creation of an original composition entitled “Resistencia en armonía.”

### Awareness campaigns

Each year, during World Antimicrobial Awareness Week (WAAW), SWICEU organized themed campus campaigns combining slogans, lectures and participatory actions. Campaigns targeted both university audiences and the broader community, addressing rational antibiotic use, prevention of infections and global AMR awareness.

These campaigns also achieved wide national media coverage, with recurring presence in print, radio, television and online outlets. (Table 3).

**Table 3:** SWICEU awareness campaigns during WAAW (2018–2025).

Year	Campaign (Spanish)	Campaign (English)	Link
2018	“Diez consejos para evitar la resistencia bacteriana a los “antibióticos”	Ten Tips to Avoid Antibiotic Resistance	https://medios.uchceu.es/actualidad‐ceu/diez‐consejos‐para‐hacer‐un‐buen‐uso‐de‐los‐antibioticos‐y‐frenar‐las‐resistencias‐bacterianas‐del‐equipo‐swiceu/
2019	“Porque no son caramelos… haz un buen uso de los antibióticos”	Because They Are Not Candy ‐ Use Antibiotics Wisely	https://medios.uchceu.es/actualidad‐ceu/porque‐no‐son‐caramelos‐haz‐un‐buen‐uso‐de‐los‐antibioticos/
2020	“En tiempos de virus, no olvides las bacterias”	In Times of Virus, Don't Forget Bacteria	https://medios.uchceu.es/actualidad‐ceu/en‐tiempo‐de‐virus‐no‐olvides‐las‐bacterias/
2021	“Esto NO es un juego, ¿juegas?”	This Is NOT a Game ‐Will You Play?	https://medios.uchceu.es/actualidad‐ceu/esto‐no‐es‐un‐juego‐el‐equipo‐swiceu‐conciencia‐jugando‐sobre‐el‐buen‐uso‐de‐los‐antibioticos/
2022	“Hazte bact‐hunter”	Become a Bact‐Hunter	https://medios.uchceu.es/actualidad‐ceu/nuevo‐reto‐swiceu‐hazte‐bact‐hunter/
2023	“Enigma en el campus”	Enigma on Campus	https://medios.uchceu.es/actualidad‐ceu/swiceu‐organiza‐un‐street‐scape‐divulgativo‐sobre‐el‐uso‐correcto‐de‐los‐antibioticos‐y‐el‐aumento‐de‐las‐infecciones‐bacterianas‐resistentes/
2024	“No te la juegues. Actúa ahora”	Act and Play. Here and Now	https://medios.uchceu.es/actualidad‐ceu/te‐puede‐tocar‐a‐ti‐nueva‐campana‐de‐swiceu‐ante‐el‐aumento‐de‐las‐infecciones‐bacterianas‐resistentes/

### Media reach and digital engagement

SWICEU's activities and materials have been disseminated through more than 50 Spanish media outlets, including both general interest and specialized platforms (e.g. ABC, La Vanguardia, Europa Press, Telecinco, COPE, BioTech Spain and Con Salud). The cumulative audience reach of this coverage exceeds 3.5 million people.

Online dissemination has been equally successful:

- The Scientific News section of CEU UCH's website has surpassed 185 000 views, becoming its most‐read content.
- On X (Twitter), posts from @SWICEU1 and @CienciaUCHCEU reached 819 000 impressions, generating over 500 000 interactions.
- The project's Instagram account (@swiceu) achieved an average of 9700 monthly impressions and 600 interactions, mainly among users aged 18–24.
- SWICEU's YouTube channel accumulated 12 843 views for educational videos and student‐produced microstories.

These metrics confirm strong engagement across both academic and public audiences, especially among youth.

### Institutional recognition and awards

Over its 8‐year trajectory, SWICEU has received seven awards: three national and four international, recognizing both its pedagogical innovation and outreach excellence.

These distinctions validate SWICEU's dual achievement: scientific and pedagogical rigor coupled with creativity and social impact.

Overall, the data demonstrate that the SWICEU model effectively combines education, research and communication. It generates measurable scientific outcomes, improves knowledge and attitudes toward antibiotics and achieves broad societal reach through innovative, gamified formats. The consistency of high satisfaction rates and learning gains confirms the project's impact as a scalable model for science education and AMR awareness worldwide.

## DISCUSSION

The results demonstrate that the SWICEU project constitutes an innovative and effective educational model for raising awareness about AMR. By combining citizen science, gamification and multimedia outreach, SWICEU has successfully engaged diverse audiences, enhanced scientific understanding and achieved significant social and media visibility. These outcomes address the persistent gap between scientific knowledge and public awareness that both the WHO and the European Commission identify as a key challenge in combating AMR.^4^, ^18^

### Citizen science and social empowerment

At the core of SWICEU lies an authentic citizen science component, where participants actively engage in microbiological research by isolating antibiotic‐producing microorganisms from soil samples. This participatory model empowers students to experience the scientific method directly, fostering critical thinking and confidence in research.^19^

By reproducing, on a smaller scale, the historical process of discovery, becoming “the new Flemings,” participants transcend passive learning. They generate real scientific data (e.g. 388 isolates showing antibiosis activity), demonstrating that citizen contributions can have tangible value for academic and industrial research alike. Similar microbiology‐based citizen science initiatives, such as Tiny Earth and MicroMundo, have shown comparable benefits in enhancing STEM motivation and understanding of AMR.^13^, ^16^, ^20^

This experiential immersion nurtures social empowerment: participants develop a realistic perception of scientific complexity, effort and uncertainty. In doing so, they build trust in scientific institutions and become better equipped to make informed decisions about antibiotic use, responding directly to the One Health approach that underpins global AMR strategies.^15^, ^21^, ^22^, ^23^

### Gamification and meaningful learning

The integration of playful strategies, from board games to musical performances, confirms the educational value of gamification in promoting motivation and conceptual retention. Previous studies have shown that game‐based learning increases engagement, facilitates complex knowledge acquisition and supports long‐term attitude change toward science and health behaviors.^24^, ^25^

In line with this evidence, SWICEU's activities, such as “2050: ¡Infección!”, Superbugs and “Hipótesis,” have proven effective in transforming abstract microbiological concepts like mutation, selective pressure and resistance transmission into experiential learning opportunities. These formats encourage collaborative reasoning, problem‐solving and empathy for the scientific process, which are central to constructivist learning theories.

The use of music and art further enriches this process by adding an emotional dimension to cognition. Educational research highlights that emotional engagement enhances memory and fosters positive health attitudes.^26^, ^27^ The “Resistencia en armonía” karaoke initiative exemplifies this, transforming a technical public health issue into a creative, emotionally resonant experience that strengthens both comprehension and dissemination through social media amplification.

By integrating creativity, fun and rigor, SWICEU validates gamification not as superficial entertainment, but as a structured pedagogical framework capable of supporting meaningful learning and scientific literacy.

### Digital reach and countering misinformation

One of SWICEU's major achievements is its successful adaptation to the digital communication ecosystem. Its presence across YouTube, Instagram and X (formerly Twitter), complemented by substantial coverage in generalistic and scientific media, allowed the project to reach more than 3.5 million people. This broad exposure is critical in an era where misinformation spreads faster than scientific evidence.^28^, ^29^

The 2024 Survey on Social Perception of Science and Technology in Spain (FECYT) confirms that audiovisual content and social media are now the main information sources on health and science, particularly among younger demographics.^7^ SWICEU's strategy, focusing on accessible, visually engaging content, aligns with these preferences, ensuring messages reach the audiences most vulnerable to misinformation.

Moreover, SWICEU's creative narratives act as counter‐discourses to pseudoscience and conspiratorial messaging. By merging factual accuracy with aesthetic appeal, these campaigns produce emotionally engaging stories that promote trust and comprehension. This approach aligns with the WHO recommendations for addressing infodemics through clear, audience‐centered communication that integrates storytelling, art and cultural adaptation.^30^

In this way, SWICEU exemplifies a model for combating health misinformation: evidence‐based, emotionally intelligent and digitally adaptive.

### Transferability and broader implications

Beyond AMR, the SWICEU methodology offers a transferable framework for addressing other public health challenges that suffer from low comprehension and high misinformation, such as vaccination, respiratory virus transmission or vector‐borne disease prevention.^31^, ^32^

Its combination of citizen science (participation), gamification (motivation) and creative media (communication/awareness) can be tailored to multiple contexts and populations.^13^, ^33^ The adaptability of its tools, board games, microstory guides, musical performances and social media campaigns makes SWICEU scalable across educational systems and cultural settings.

This integrative approach echoes the principles of Responsible Research and Innovation (RRI), which call for inclusive, reflective and anticipatory science communication that connects citizens with researchers and policymakers.^34^, ^35^ By transforming students into communicators and citizens into co‐creators, SWICEU contributes to building a scientifically literate and socially responsible public.

### Limitations and future directions

While the project demonstrates clear educational and communication outcomes, certain limitations should be noted. First, most of the evaluative data are short term, measuring immediate knowledge gains and satisfaction; longitudinal studies would help determine the persistence of behavioral change.

Second, expanding the evaluation of the effectiveness of the gamification strategies implemented is recommended through the inclusion of focus groups and structured gameplay sessions analyzed using multiple observational methodologies. Such an approach would facilitate the generation of robust, specific and comparable evidence across the educational games on antimicrobial resistance (AMR) developed by the SWICEU team.

Third, separating the evaluation of different gamification tools by demographic variables (age, gender, prior knowledge) could yield further insights into which formats are most effective for specific audiences.

Finally, although social media analytics provide valuable insight into reach and engagement, future work could employ deeper sentiment and network analysis to evaluate message diffusion and influence.

Future research will explore these areas through mixed‐methods designs and international collaborations, leveraging SWICEU's repository of open‐access materials.

## METHODS

### Study design and participants

The SWICEU project was implemented annually from 2017 to 2025 as a long‐term citizen science and educational program aimed at raising awareness about AMR among pre‐university and university students.

A total of 853 pre‐university students between 14 and 16 years old from seven secondary schools and 287 university students from CEU Cardenal Herrera University participated in the eight editions analyzed in this study. Participants were aged between 14 and 24 years and represented diverse socioeconomic and geographical backgrounds across the Valencian Community.

The project integrated both laboratory‐based scientific participation and creative outreach activities, combining experiential learning with gamified and creative communication strategies. Each academic year, participants were introduced to fundamental microbiology concepts, performed laboratory experiments to identify antibiotic‐producing microorganisms and later developed and evaluated outreach products (e.g., games, microstories, videos) to communicate AMR concepts to broader audiences.

### Educational and laboratory component

The experimental phase focused on discovering potential antibiotic‐producing microorganisms from local soil samples. Pre‐university students, guided by university mentors, collected and processed samples, isolated colonies and tested them for antibiosis.

The scientific impact of this component was evaluated based on three main quantitative parameters: number of analyzed soil samples, number of isolated microorganism colonies and number of isolates exhibiting antibiosis activity.

In addition, a dual knowledge and satisfaction survey was administered to all pre‐university and university participants, as well as to accompanying teachers.

To assess knowledge acquisition, a 12‐item multiple‐choice questionnaire was designed collaboratively by SWICEU lecturers and participating students. It addressed core concepts on antibiotic use and resistance (e.g. mechanisms of resistance, misuse consequences, infection prevention). The same questionnaire was administered before and after participation in the project to evaluate learning gains. Each question had four possible answers, with only one correct (Supplementary material 1).

The survey was available in both Spanish and English, and all responses were collected anonymously to ensure confidentiality and reduce bias. Pre‐ and post‐test data allowed measurement of individual and group‐level improvement in AMR literacy.

Participant satisfaction was measured via structured Likert‐scale surveys evaluating several dimensions: scientific interest, antibiotic resistance, personal opinion about the project and best and worst aspects of the activity (Supplementary materials 2 and 3).

### Gamification and outreach component

In parallel with laboratory work, the SWICEU team developed multiple gamification and creative communication resources to enhance learning and outreach impact. These included board games, educational scape boxes, Cluedo‐style mystery games, the “Create Your Microstory” narrative guide, scientific karaoke and music videos and infographic bookmarks and digital storytelling tools.

These resources were designed iteratively based on feedback from previous editions, ensuring continuous refinement. Activities were tailored to specific audiences, secondary students, university participants or community members, according to their educational level and context.

Each year, a final on‐site outreach event was organized at CEU UCH, where participants presented their creative outputs, played the games and shared microstories. During these events, gamified materials were distributed and evaluated through direct observation and participant feedback.

### Evaluation of public awareness and media impact

The impact of SWICEU's outreach campaigns on public awareness of AMR was assessed through media and digital analytics.

Quantitative data were obtained from official SWICEU social media accounts (YouTube, Instagram, X/Twitter), using built‐in analytics to measure: followers and reach, number of posts, engagement (likes, comments, shares) and video views and watch time.

Qualitative data were collected by the Scientific Culture Unit (UCC + i) of CEU UCH, which systematically archived press releases, news coverage and online dissemination from external scientific communication platforms. The type of media (academic, educational, journalistic or institutional) and their reach were categorized for content analysis.

This approach enabled triangulation of data between internal analytics and external media visibility, providing a comprehensive picture of community engagement.

### Evaluation of scientific career motivation

To assess whether participation increased students' interest in scientific careers, becoming the “new Flemings” needed for the future, several indicators were analyzed:

- Attendance, participation and quality of oral and poster presentations delivered by pre‐university students at the final annual event.
- Number of university student presentations, posters and communications related to SWICEU and MicroMundo at the Spanish Society for Microbiology (SEM) Congress.
- Self‐reported motivation and intention to pursue scientific studies, collected through post‐project surveys and open comments.

These indicators provided both direct and indirect measures of motivational impact and perceived self‐efficacy in science learning (Table 4).

**Table 4:** Evaluation instruments of SWICEU project.

Instrument	Purpose	Type of Data	Participants	Frequency
Pre/post knowledge survey	Measure understanding of AMR and antibiotics	Quantitative (scores, correct answers)	Pre‐university and university students	Twice per edition
Satisfaction survey	Evaluate perceived quality, enjoyment and relevance	Quantitative (Likert scale 1–5) + qualitative comments	All participants and teachers	End of activity
Social media analytics	Assess public engagement and outreach impact	Quantitative (reach, engagement)	General audience	Continuous
Event feedback & interviews	Assess emotional and motivational engagement	Qualitative	Students, teachers, visitors	Annual
Media coverage analysis	Assess dissemination and external recognition	Mixed (content + frequency)	General audience	Annual

### Data analysis

Quantitative survey results were analyzed using descriptive statistics and comparative analysis between pre‐ and post‐intervention scores. Mean values, standard deviations and percentage of correct responses were calculated to determine knowledge gains.

Qualitative data from open responses, interviews and participant reflections were coded thematically to identify patterns in motivation, emotional engagement and perceived learning outcomes. Media and social media data were aggregated to estimate overall reach and engagement.

Where applicable, analyses were disaggregated by participant type (pre‐university vs. university) and activity type (laboratory, gamification or outreach).

### Ethical considerations

All procedures involving human participants were conducted in accordance with institutional ethical standards and the Declaration of Helsinki.

Participation was voluntary; informed consent was obtained from all participants (and guardians when applicable), and data were processed anonymously. The educational and outreach activities were developed under the university's SWICEU project, ensuring alignment with RRI principles.

This mixed‐methods design allowed a comprehensive evaluation of SWICEU's educational, communicative and motivational impact. By integrating laboratory research, creative outreach and systematic evaluation, the project provides an evidence‐based model for participatory science communication on AMR and other global health challenges.

## CONCLUSIONS

Universities and research centers remain the most trusted institutions to communicate how scientific and technological advances affect society. The information disseminated through these sources is generally perceived as credible and understandable. However, current global challenges such as AMR demand that academic institutions go beyond conventional outreach, developing innovative formats capable of reaching broader and more diverse audiences with accurate, engaging and evidence‐based content.

The SWICEU project, promoted by CEU Cardenal Herrera University, represents a successful and replicable model that integrates citizen science, gamification and creative communication to increase public awareness and understanding of AMR. By combining authentic scientific research with playful, participatory learning, SWICEU bridges the traditional gap between the laboratory and society. The project has demonstrated that science communication can be both rigorous and accessible when it is emotionally engaging, interdisciplinary and grounded in real participation.

The results obtained throughout its eight editions confirm that gamified and creative learning strategies not only motivate participation but also improve conceptual retention, foster curiosity about scientific careers and encourage responsible behavior regarding antimicrobial use. The significant improvements in knowledge scores among pre‐university students, the satisfaction expressed by participants and the project's wide media impact all points to the effectiveness of this integrated approach.

Moreover, the project illustrates how citizen science can serve as a vehicle for empowerment: by involving students and educators as active contributors to antibiotic discovery, SWICEU transforms them into “new Flemings,” citizens who understand, value and disseminate the scientific process itself. This sense of shared responsibility reinforces trust in science and promotes informed decision‐making in public health.

In the face of the “silent pandemic” of AMR, gamification and creative science communication emerge as essential allies. SWICEU's approach shows that playful learning does not trivialize complex topics; rather, it transforms them into memorable, emotionally resonant experiences that sustain attention and understanding. Through this strategy, the project counters misinformation, strengthens scientific literacy and connects diverse audiences with the relevance of microbiology and antibiotic stewardship.

Looking ahead, the transferability of this model extends well beyond AMR. The integration of experiential learning, artistic expression and digital dissemination can be adapted to other global health priorities, such as vaccination, emerging infections or environmental health, where comprehension and behavioral change are equally critical. Developing digital and hybrid versions of SWICEU's activities could further expand its international reach, facilitating collaboration across institutions and cultures.

Ultimately, the success of SWICEU underscores a broader conclusion: bridging research, communication and citizen participation is not supplementary to science; it is integral to it. When universities lead in creating participatory, creative and evidence‐based communication, they transform education into collective action. In doing so, they help build a society that not only understands science but also contributes actively to its advancement and application in confronting the pressing health challenges of our time.

## FINAL REMARKS

The story of the “new Flemings” is not only an educational experiment but also a movement of young people learning to understand, communicate and defend antibiotics, the cornerstone of modern medicine. By singing, playing and experimenting, they echo the legacy of Fleming in the language of a new generation.

SWICEU stands as evidence that when science is lived with curiosity and creativity, its message resonates beyond laboratories and classrooms, into society itself.

## AUTHOR CONTRIBUTIONS

**Beatriz Suay‐García:** Investigation; methodology; writing – review and editing. **Elisa Marco‐Crespo:** Investigation; methodology; writing – review and editing. **María‐Teresa Pérez‐Gracia:** Conceptualization; funding acquisition; writing – original draft; investigation; methodology; validation; visualization; writing – review and editing; data curation; supervision; resources; project administration; formal analysis. **Antonio Tarín‐Pelló:** Writing – original draft; investigation; methodology; writing – review and editing; data curation; formal analysis. **José Ignacio Bueso‐Bordils:** Investigation; methodology; writing – review and editing. **Sara Fernández‐Álvarez:** Investigation; methodology; writing – review and editing. **Carolina Galiana‐Roselló:** Investigation; methodology; writing – review and editing.

## CONFLICT OF INTEREST

The authors declare no conflicts of interest.

## Supporting information


Supplementary material 1

Supplementary material 2

Supplementary material 3

## Data Availability

All datasets and supplementary materials (educational guides, gamification tools, videos and evaluation instruments) are available through the SWICEU project's open repository: https://medios.uchceu.es/actualidad-ceu/etiqueta/swiceu/; https://www.youtube.com/playlist?list=PLDt-M0gErNryEdJVk9QvGhX2GMM9UFQSu; https://www.youtube.com/playlist?list=PLDt-M0gErNrzKnKU9FJvlUtRuURX0nSBm; https://x.com/SWICEU1.

## References

[1] Tarín‐Pelló A , Suay‐García B , Pérez‐Gracia MT . Antibiotic resistant bacteria: current situation and treatment options to accelerate the development of a new antimicrobial arsenal. Expert Rev Anti Infect Ther 2022; 20: 1095–1108. 35576494 10.1080/14787210.2022.2078308

[2] Murray CJL , Ikuta KS , Sharara F , *et al*. Global burden of bacterial antimicrobial resistance in 2019: a systematic analysis. Lancet 2022; 399: 629–655. 35065702 10.1016/S0140-6736(21)02724-0 PMC8841637

[3] Peñalva G , Cantón R , Pérez‐Rodríguez MT , *et al*. Burden of bacterial antimicrobial resistance among hospitalised patients in Spain: findings from three nationwide prospective studies. Lancet Reg Health Eur 2025; 51: 101220. 39958398 10.1016/j.lanepe.2025.101220 PMC11830305

[4] World Health Organization . Antimicrobial resistance global surveillance report 2024. https://www.who.int/publications/i/item/9789241509763. Accessed December 19, 2025.

[5] Abbas A , Barkhouse A , Hackenberger D , Wright GD . Antibiotic resistance: a key microbial survival mechanism that threatens public health. Cell Host Microbe 2024; 32: 837–851. 38870900 10.1016/j.chom.2024.05.015

[6] Special eurobarometer 522 antimicrobial resistance report fieldwork: February‐March 2022. https://europa.eu/eurobarometer/surveys/detail/2632. Accessed December 19, 2025.

[7] Fundación Española para la Ciencia y la Tecnología . Encuesta de percepción social de la ciencia y la tecnología en España (EPSCT) 2024. 2025 10.58121/90E2-ME77.

[8] European Comission . Special Eurobarometer SP557: European citizens' knowledge and attitudes towards science and technology. February 2025. https://europa.eu/eurobarometer/surveys/detail/3227. Accessed December 19, 2025.

[9] Castro‐Sánchez E , Garelick H , Pérez‐Gracia MT , Aminov R . The role of education in raising awareness towards antimicrobial resistance (AMR). Front Microbiol 2024; 15: 1444502. 38989026 10.3389/fmicb.2024.1444502 PMC11233767

[10] Smith H , Allf B , Larson L , *et al*. Leveraging citizen science in a college classroom to build interest and efficacy for science and the environment. Citizen Sci Theory Pract 2021; 6: 29.

[11] Tahlil KM , Nwaozuru U , Conserve DF , *et al*. Crowdsourcing to support training for public health: a scoping review. PLOS Global Public Health 2023; 3: e0002202. 37494311 10.1371/journal.pgph.0002202 PMC10370701

[12] Bueso‐Bordils JI , Suay‐García B , Galiana‐Roselló C , Marco‐Crespo E , Pérez‐Gracia MT . Evaluation of the impact of the tiny earth project on the knowledge about antibiotics of pre‐university students in the province of Valencia on three different school years (2017–2020). Front Microbiol 2020; 11: 576315. 33329439 10.3389/fmicb.2020.576315 PMC7710527

[13] Gil‐Serna J , Antunes P , Campoy S , *et al*. Citizen science to raise antimicrobial resistance awareness in the community: the MicroMundo project in Spain and Portugal. J Microbial Biotechnol 2025; 18: e70123. 10.1111/1751-7915.70123 PMC11878116 40035803

[14] Davis E , Sloan T , Aurelius K , *et al*. Antibiotic discovery throughout the small world initiative: a molecular strategy to identify biosynthetic gene clusters involved in antagonistic activity. Microbiologyopen 2017; 6: e00435. 28110506 10.1002/mbo3.435 PMC5458470

[15] Tarín‐Pelló A , Marco‐Crespo E , Suay‐García B , Galiana‐Roselló C , Bueso‐Bordils JI , Pérez‐Gracia MT . Innovative gamification and outreach tools to raise awareness about antimicrobial resistance. Front Microbiol 2022; 13: 977319. 36187952 10.3389/fmicb.2022.977319 PMC9520167

[16] Tarín‐Pelló A , Suay‐García B , Marco‐Crespo E , Galiana‐Roselló C , Bueso‐Bordils JI , Pérez‐Gracia MT . Evaluation of knowledge about antibiotics and engagement with a research experience on antimicrobial resistance between pre‐university and university students for five school years (2017‐2021). Front Microbiol 2022; 13: 959187. 36033886 10.3389/fmicb.2022.959187 PMC9402252

[17] Pérez Gracia M , Bueso Bordils J , Galiana‐Roselló C , Marco‐Crespo E , Suay‐García B . Create your microstory: guidelines for microbe inspired microstories. 2024 https://hdl.handle.net/10637/23390.

[18] European Commission. 2024. https://health.ec.europa.eu/antimicrobial‐resistance/eu‐action‐antimicrobial‐resistance_en?utm_source=chatgpt.com.

[19] Ahannach S , Van Hoyweghen I , Verhoeven V , Lebeer S . Citizen science as an instrument for women's health research. Nat Med 2024; 30: 3445–3454. 39578585 10.1038/s41591-024-03371-2

[20] Piroddi C , Gilby L , Koivusalo M , McCallum A , Brown A , Stephenson C . The hegemony of far‐right populism, project 2025, and the dangers ahead for science and public health. Int J Soc Determinants Health Health Serv 2025; 56: 27551938251367853. 10.1177/27551938251367853 PMC12662832 40820411

[21] Hurley A , Chevrette MG , Acharya DD , *et al*. Tiny earth: a big idea for STEM education and antibiotic discovery. MBio 2021; 12: e03432‐20. 33593964 10.1128/mBio.03432-20 PMC8545114

[22] Plechatá A , Makransky G , Böhm R . A randomized controlled trial investigating experiential virtual reality communication on prudent antibiotic use. NPJ Digit Med 2024; 7: 244. 39266716 10.1038/s41746-024-01240-3 PMC11392957

[23] Mitchell J , Arjyal A , Baral S , King R , Manandhar S , Cooke P . Can community engagement occur online: a framework analysis of pandemic‐induced changes to a project on antimicrobial resistance (AMR). J Public Health Emerg 2025; 9: 14.

[24] Sharmin N , Shah M , Chow AK . Exploring students' experience with game‐based learning: a descriptive study. Can J Dent Hyg 2025; 59: 98–106. 40809529 PMC12341505

[25] Dillon MJ , Edwards J , Hughes A , Stephenson HN . Beyond lectures: leveraging competition, peer discussion and real‐world scenarios in a digital card game to enhance learning of microbiology and immunology concepts. Access Microbiol 2025; 7: 000900.v3. 10.1099/acmi.0.000900.v3 PMC11825987 39959471

[26] Salakka I , Pitkäniemi A , Pentikäinen E , Saari P , Toiviainen P , Särkämö T . Emotional and musical factors combined with song‐specific age predict the subjective autobiographical saliency of music in older adults. Psychol Music 2024; 52: 305–321. 38708378 10.1177/03057356231186961 PMC11068497

[27] Salakka I , Pitkäniemi A , Pentikäinen E , *et al*. What makes music memorable? Relationships between acoustic musical features and music‐evoked emotions and memories in older adults. PLoS One 2021; 16: e0251692. 33989366 10.1371/journal.pone.0251692 PMC8121320

[28] Molan K , Zore A , Kregar Velikonja N . Health literacy and interventions on antibiotics use and AMR in younger generations in high‐income countries—a systematic review. Antibiotics 2025; 14: 940. 41009918 10.3390/antibiotics14090940 PMC12466577

[29] Health in the age of disinformation. Lancet 2025; 405: 173. 39826957 10.1016/S0140-6736(25)00094-7

[30] Combrink HMVE , Mkungeka P . Misfluencers, the human agents behind AI‐driven Infodemics. J Health Commun 2025; 29: 1–7. 10.1080/10810730.2025.2538529 40729948

[31] Ferreira Caceres MM , Sosa JP , Lawrence JA , *et al*. The impact of misinformation on the COVID‐19 pandemic. AIMS Public Health 2022; 9: 262–277. 35634019 10.3934/publichealth.2022018 PMC9114791

[32] Jin SL , Kolis J , Parker J , *et al*. Social histories of public health misinformation and infodemics: case studies of four pandemics. Lancet Infect Dis 2024; 24: e638–e646. 38648811 10.1016/S1473-3099(24)00105-1

[33] Schaller S , Schumann C , Wolling J . Public engagement by strategic science communication: an evaluation study on the impact of an interactive science exhibition on the German energy transition. Front Commun 2025; 10: 1657572.

[34] Ivani S , Dutilh Novaes C . Public engagement and argumentation in science. Eur J Philos Sci 2022; 12: 54. 35958803 10.1007/s13194-022-00480-y PMC9361237

[35] Bidstrup MV , Gabova S , Kilintzis P , *et al*. The RRI citizen review panel: a public engagement method for supporting responsible territorial policymaking. J Innov Entrep 2024; 13: 2.

