# Supplementary material for: The new Flemings now sing: a methodological evaluation of gamification and citizen science strategies to raise awareness on antimicrobial resistance
Source: Immunol Cell Biol. 2026 Feb 13;104(3):265–75. doi: 10.1111/imcb.70094 (PMC12972230; doi:10.1111/imcb.70094)
Supplement: Supplementary file 1 — Supplementary material 1 [file IMCB-104-265-s003.pdf]

ANTIBIOTIC KNOWLEDGE SURVEY

SWICEU Project

This survey allows us to know what is your knowledge on antibiotics and their use.
The data you provide is anonymous and confidential.
Circle (A) the right answer.

1. What is the cause for the common cold??
A. Virus
B. Bacteria
C. Getting cold
D. Having your hair wet for a long time

2. Is a prescription necessary to buy antibiotics?
A. Yes, it is always necessary.
B. No, only sometimes (when I have something serious or I do not know what I have)
C. No, a prescription is never necessary.

3. Which of the following drugs is an antibiotic?
A. Aspirin
B. Amoxicillin
C. Paracetamol
D. Ibuprofen

4. Are antibiotics useful for any occasion?
A. Yes, they can be used to fight any type of microorganism.
B. No, only for infections caused by bacteria, fungi and some parasites.
C. No, only for infections caused by virus such as the flu.
D. Yes, but it depends on the age of the person infected and if they already have a disease.

5. Can a bacterial infection be treated with any antibiotic?
A. Yes
B. No
C. Depends on the age
D. Depends on the stage at which it was diagnosed

6. Which indications should be considered when taking antibiotics?
A. Medical instructions (schedule and dose) should be followed rigorously.
B. Treatment should be suspended as soon as symptoms disappear.
C. It is important to keep the prescription in order to use it in the future, if the same symptoms appear.
D. All of the above.

7. If we do not take antibiotics correctly:

A. We will get better.
B. We will have problems with the antibiotic resistance that bacteria will probably develop.
C. Animals will become resistant to antibiotics and will never be infected by bacteria.
D. We will use other drugs that "kill bacteria" such as paracetamol, ibuprofen and omeprazole.

8. What is antibiotic resistance?

A. Antibiotic resistance occurs when microorganisms (bacteria, virus, fungi or parasites) undergo changes, which make the drugs used to treat infections ineffective. (WHO Definition)
B. Any mechanism produced by the human body that degrades these drugs, rendering them ineffective and, therefore, lacking therapeutic effect.
C. Processes carried out by bacteria and humans jointly that cause the antibiotic to lose its efficacy for the infection we want to treat.
D. None of the above.

9. Antibiotic resistant bacteria can be transmitted to humans:

A. Through contact with someone who has an infection caused by antibiotic resistant bacteria.
B. Through contact with something touched by a person who has an infection caused by antibiotic resistant bacteria. (for example, in hospitals with deficient hygiene, the hands of health workers or the instruments they use)
C. Through contact with animals, food or water carrying antibiotic resistant bacteria.
D. All of the above.

10. Which of the following is essential in combating antibiotic resistance?

A. Vaccination
B. Taking antibiotics only under medical prescription, at the correct dose and during the necessary time.
C. Investing in the research of new antibiotics.
D. All of the above.

11. Mark the incorrect statement:

A. I can always recommend someone I know a treatment that I used to treat a bactreial infection. Furthermore, if I had leftover medication, it would be correct to share it with them in order to reduce health expenses.
B. I should never recommend/lend an infection treatment to anyone. In fact, if I had leftovers, I should take them to the nearest pharmacy so they can dispose of them in the correct way.
C. I should never take antibiotics for a viral infection such as the flu or a cold.
D. Even if symptoms have disappeared, I will never stop taking antibiotics halfway through the treatment, seeing as that would favor the appearance of antibiotic resistances.

12. According to the World Health Organization (WHO), in 2050 it is estimated that the first cause of death will be:

A. Cancer
B. Infections caused by antibiotic resistant bacteria
C. Cardiovascular disease, such as stroke or ictus
D. Neurodegenerative disease, such as Alzheimer's disease

THANK YOU FOR YOUR COLLABORATION!
