# Supplementary material for: The new Flemings now sing: a methodological evaluation of gamification and citizen science strategies to raise awareness on antimicrobial resistance
Source: Immunol Cell Biol. 2026 Feb 13;104(3):265–75. doi: 10.1111/imcb.70094 (PMC12972230; doi:10.1111/imcb.70094)
Supplement: Supplementary file 2 — Supplementary material 2 [file IMCB-104-265-s002.pdf]

STUDENT SATISFACTION QUESTIONNAIRE
SWICEU Project

Teachers involved in the *Small World Initiative/MicroMundo* project, during the 2024-2025 academic term by Universidad CEU Cardenal Herrera, want to collect the opinion of school teachers about the development of this initiative in order to evaluate the degree of fulfillment of the established goals. Your colaboration by completing this questionnaire will provide valuable information in order to improve this project.

The data you provide is anonymous and will be treated confidentially. We, therefore, ask you to answer sincerely.

Evaluate from 1 to 5 the different sections, knowing that 1 is the most negative and 5 the most positive.

		1	2	3	4	5
**Scientific Interest**	Participating in this project has increased your scientific curiosity					
	You consider that this experience has brought you closer to a real problem					
	You believe your results can contribute to scientific progress					
	Evaluate the repercussion of this experiment on the knowledge of microbial diversity in nature					
**Antibiotic Resistance**	Your participation has contributed to a better understanding of the issue regarding antibiotic resistance					
	This experience has changed your perception on antibiotic use					
	This project has contributed to your knowledge of antibiotic resistance					
**Others**	Reflect your global opinion about participating in this project					
	This project has improved your scientific knowledge					
	How do you evaluate your experience working on a real problem?					
	Would you recommend other students or schools to participate in this project?					

**BEST ASPECT OF THE ACTIVITY**
**WORST ASPECT OF THE ACTIVITY**
**IMPROVABLE ASPECTS OF THE ACTIVITY**

THANK YOU FOR YOUR COLLABORATION!
