# Supplementary material for: The new Flemings now sing: a methodological evaluation of gamification and citizen science strategies to raise awareness on antimicrobial resistance
Source: Immunol Cell Biol. 2026 Feb 13;104(3):265–75. doi: 10.1111/imcb.70094 (PMC12972230; doi:10.1111/imcb.70094)
Supplement: Supplementary file 3 — Supplementary material 3 [file IMCB-104-265-s001.pdf]

# TEACHER SATISFACTION QUESTIONNAIRE
# SWICEU Project

Teachers involved in the *Small World Initiative/MicroMundo* project, during the 2024-2025 academic term by Universidad CEU Cardenal Herrera, want to collect the opinion of school teachers about the development of this initiative in order to evaluate the degree of fulfillment of the established goals. Your colaboration by completing this questionnaire will provide valuable information in order to improve this project.

**The data you provide is anonymous and will be treated confidentially.** We, therefore, ask you to answer sincerely.

**Evaluate from 1 to 5 the different sections, knowing that 1 is the most negative and 5 the most positive.**

| | | 1 | 2 | 3 | 4 | 5 |
|---|---|---|---|---|---|---|
| **Evaluation of learning and interest of students** | Taking part in this project has awakened scientific curiosity/interest in students | | | | | |
| | This project has improved the scientific knowledge of students | | | | | |
| | This project has helped students acquire practical laboratory skills | | | | | |
| | Students have understood the problem regarding antibiotic resistance | | | | | |
| | Students have understood the microbial diversity existing in nature | | | | | |
| | Students have easily understood what was explained to them | | | | | |
| | Students were able to carry out the practical work easily | | | | | |
| **Personal opinion about the project** | Reflect your global opinion about this project | | | | | |
| | Reflect your opinion about the work carried out by the team in charge of the project at your school | | | | | |
| | Would you recommend other schools to take part in this project | | | | | |

**BEST ASPECT OF THE ACTIVITY**

**WORST ASPECT OF THE ACTIVITY**

**ASPECTS THAT COULD BE IMPROVED**

**THANK YOU FOR YOUR COLLABORATION!**
